# Hydrastin in Laryngeal Phthisis

**Published:** 1883-02

**Authors:** 


### Hydrastin in Laryngeal Phthisis.

Dr. Bird (*Australian Med. Jour.*) claims good results from the treatment of laryngeal phthisis with a spray composed of hydrastin, glycerine, borax and morphia. A combination of this kind would seem likely to be of advantage.—*Ibid.*

